# Commentary on Henrik Walter's “The third wave of biological psychiatry”

**DOI:** 10.3389/fpsyg.2013.00832

**Published:** 2013-11-20

**Authors:** Markus R. Pawelzik

**Affiliations:** EOS-Klinik für PsychotherapieMuenster, Germany

**Keywords:** biologism in psychiatry, zombie-psychology, medical model of mental disorder, psychological individualism, behavioral self-programing of the brain

There is good news: “Biological psychiatry is no longer biologistic!” According to Walter (2013), a seasoned German neuroscientist, psychiatrist and philosopher, a putative “third wave” of biological psychiatry has overcome many flaws that for a long time motivated our opposition to biological psychiatry: The third wave is no longer reductionist, localizations, or ignorant toward the normative, social, and cultural dimensions of mental problems. Rather, it analyses dynamic neural network activities, distinguishes multiple levels of description and takes every kind of context dependence you name into account. Furthermore, the third wave is aware of its methodological, theoretical and therapeutic limitations; and it self-criticizes all kinds of programmatic overstatements common in the field. In a nutshell: The third wave liberated biological psychiatry from its biologistic roots. It restricts itself to the legitimate search for the “biosignatures” of mental disorders.

From a down-to-earth point of view, this is hard to believe. Participate in any conference of biological psychiatrists these days and you can see that big money, pharmaceutical industry, reductionist ideas of man and a biologically biased psychiatric practice form a veritable, obviously flourishing coalition. But blaming theory for failed praxis would be unfair to Walter's informed and interesting paper. The question put forward by my commentary is, therefore, a theoretical one: *Are there any crucial remnants of biologism in Walter's apology of third wave biological psychiatry?* I think there are at least two points that justify further critical examination—points that Walter belabors extensively: the role of the mind and the role of the medical model of mental disorders in biological psychiatry.

*What about the mind?* Mentally ill people suffer. They consciously experience the burden of their condition. Psychiatry always intended to deal with the “mindedness” of mentally disordered people. But can modern neurosciences—the foundational basis of biological psychiatry—explain the phenomenon of mental life? The issue is not that mindedness depends on brain activity; it does. The controversial subject is that our scientific approaches to the study of the mental realm are misguided. Let me hint at three exemplary ways of misguidance:

- Due to the methodological restrictions of the behavioral sciences, biological psychiatry studies *“zombies”*—people with the same neuro-behavioral properties but without subjective consciousness. Zombie-psychology looks for strict correlations between operationalized behavioral paradigms and objectively measured neuronal activities; proceeding in this way excludes the mental realm by definition. As long as this is the case, all that biological psychiatry can ask for is the special status of an “applied clinical neuroscience” assisting general psychiatry.
- Even worse is the *individualism* of biological psychiatry: The disturbed mental functions of psychiatric patients are regarded as individual, natural dispositions of the brain. Being a naturalist myself, I strongly disagree with this premise: Mental functions—our abilities to feel, to think, to act—are collectively defined, socio-cultural artifacts rather than purely natural, individual dispositions. To acculturate an individual, the “natural bottom up processes” of our species-design are developmentally coupled with socially mediated “non-natural top down processes” (Prinz, 2012). Conventional “mental instruments” are a result of the adaption to the cognitive niches of our culture (Sterelny, 2003); and they have to be continuously interpersonally re-calibrated to be effective (Pawelzik, 2013a). Walter acknowledges this point when he argues for a multi-level approach: The influence of interpersonally mediated cultural influences takes place on *supra-individual levels* of analysis: Activities on all levels—from gene expression to the cultural scaffolding of behavior—are relevant to understand a mind. But biological psychiatry does not study singular developments; it aims at the regularities of mental disorders; it therefore has to specify *which conditions on what levels generate the syndromatic pattern* that defines a kind of disorder. Looking into the brain for “biosignatures” will not inform you about the impact of the supra-individual level processes. In personal communication Walter would rebut: “All inputs, despite the level of origin, converge on processes in the brain; the brain is the eye of the needle of pathogentic influences.” But what about the interpretation of the data you gather from the needle's eye? Will you be able to understand them if you disregard the nested senso-motoric slopes that “embody,” “embed,” and “extend” the “enactive” mind in a body, in a situation and a culture? Since Walter doesn't give us the slightest idea how this might succeed, I take his third wave as an individualistically limited enterprise.
- What about the mind's *active role* in the etiology of pathological behavior? Walter mentions Levy's 2013 argument that addiction is no brain disorder like Alzheimer's since addicts can stop their addicted behavior. He counters this argument with the example of phenylketonuria: this metabolic disorder does not stop when you put your child on a phenylketon-free diet, since the pathogenetic mechanism is still left unchanged. Following this line of argument, one could say: all my thoughts, decisions and intentional actions—my way of life—will not change my brain, since the pathogenetic biological predispositions—from risk genes to temperament and maladaptive schemata—are still left unchanged. But this is simply not true. My actions can successively change my brain and its pathogenetic potentials. My strategies of *effortful control of attention*, e.g., that were entrained in early attachment-interactions and are actively developed to deal with all kinds of practical and social challenges in later life have an enormous influence on my behavior (Posner, 2012)—and therefore on my risk to develop a mental disorder (Pawelzik, 2013b). If the mind that supervenes on brain states can actively change brain states, thereby redirecting the brain's development depending on various environmental contingencies—than this “enactive mind” is obviously underspecified by the third wave concepts Walter offers. In order to overcome its traditional “mindlessness,” biological psychiatry will have to undergo nothing less than a conceptual revolution.

*Psychiatry is mainly about mental disorders, not about mindedness.* “You are asking for too much,” a sympathetic biological psychiatrist might respond. Scientific psychiatry would overstretch its chances if it tried to focus on the mindedness of the mentally ill. Scientific psychiatry's role is first and foremost to define and analyze mental disorders and to develop effective therapies. No wonder that Walter spilled most of his ink on the regulative idea of the field—the idea that mental disorders as nomological kinds.

“According to the third wave of biological psychiatry, mental disorders are relatively stable prototypical, dysfunctional patterns of experience and behavior,” Walter declares. But is this really the case outside of university departments (where patients are strictly selected to fit scientific study designs)? Most of my patients show syndrome shifts, present symptoms that fit multiple diagnoses (on all axis of the DSM-taxonomy) and suffer from a wide range of nosologicaly ignored problems. These facts obviously limit the “prototypicality” of their illness. Furthermore, the contingencies of their learning histories, the influence of transdiagnostic, i.e., disease-unspecific developmental trajectories like attachment organization, their social situation and their individual “identity politics” call for an individualizing behavioral analysis that stretches over the whole spectrum of descriptive levels in order to plan effective therapy. No wonder that third wave biological psychiatry did not show up with the most convincing proof for the role of nomological pathogenesis—a therapy that fixes the pathogenetic mechanism.

Nevertheless, Walter might answer, that the phenotypical heterogeneity of mental illness might still depend on relatively homogenous biological regularities. Let's take genetic risk factors, e.g., Colleagues of Walter just demonstrated that *the same* genetic risk loci of two calcium channel signaling genes are involved in the development of five major mental disorders—autism, attention deficit-hyperactivity disorder, bipolar disorder, major depressive disorder and schizophrenia—that make up an astonishing broad spectrum of psychopathologies (Cross-Disorder Group of the Psychiatric Genomics Consortium, 2013). The pleiotropic effects of CACNA1C and CACNB2, this study highlights, might be due to the susceptibility for specific phenotypes depending on differential environmental influences. Well, we know that mental disorders are of multi-genetic origin and that gene-x-environment-interactions play an important role. But this truism doesn't specify the un-numberable interactive possibilities of epigenetics. To defend the idea of a quasi-nomologic etiology of mental disorder, one should at least be able to determine the interaction of a number of “risk-genes” that generate disease-specific “endophenotypes.” In the case of Major Depressive Disorder, for instance, experts are discussing a rather long list of potential candidates ranging from anomalies of the HPA-axis to decreased subgenual PFC-activity (Hasler et al., 2004). What if vague syndromes like depression consist of individual mixtures of “sub-endophenotypes?” That might be the case; therefore we have to find out on which level we find the “mechanistic property clusters” that distinguish between supposed types of mental disorders, Walter might answer. If the nomological structure is not found on the levels of epigenetics or proteomics, it might still be found on the levels of the connectome and/or the activation patterns of definable neural networks. All we need is a biotype that robustly correlates with certain experiential and behavioral patterns. Without going into further details, my question is: What will happen if no connective or functional patterns fit our established nosology? Will we go for a better, strictly biological nosology, as Thomas Insel demands? Or will the regulative idea of psychiatry—mental disorders are nomological kinds—slowly degenerate? I don't know. But as a keen observer of the dynamic market of “biosignatures” I wouldn't put much money on this meta-hypothesis that Walter's third wave still entertains.

To sum up: Walter's description of third wave biological psychiatry is on the right track: We should embrace his purgation of a lot of biologistic thought. Still, as I tried to show, Walter left the main conceptual pillars of biological psychiatry—“mindlessness” and “medical model”—basically untouched.
